# Rapid, Precise, and Accurate Counts of *Symbiodinium* Cells Using the Guava Flow Cytometer, and a Comparison to Other Methods

**DOI:** 10.1371/journal.pone.0135725

**Published:** 2015-08-20

**Authors:** Cory J. Krediet, Jan C. DeNofrio, Carlo Caruso, Matthew S. Burriesci, Kristen Cella, John R. Pringle

**Affiliations:** Department of Genetics, Stanford University School of Medicine, Stanford, California, United States of America; American University in Cairo, EGYPT

## Abstract

In studies of both the establishment and breakdown of cnidarian-dinoflagellate symbiosis, it is often necessary to determine the number of *Symbiodinium* cells relative to the quantity of host tissue. Ideally, the methods used should be rapid, precise, and accurate. In this study, we systematically evaluated methods for sample preparation and storage and the counting of algal cells using the hemocytometer, a custom image-analysis program for automated counting of the fluorescent algal cells, the Coulter Counter, or the Millipore Guava flow-cytometer. We found that although other methods may have value in particular applications, for most purposes, the Guava flow cytometer provided by far the best combination of precision, accuracy, and efficient use of investigator time (due to the instrument's automated sample handling), while also allowing counts of algal numbers over a wide range and in small volumes of tissue homogenate. We also found that either of two assays of total homogenate protein provided a precise and seemingly accurate basis for normalization of algal counts to the total amount of holobiont tissue.

## Introduction

In studies of animal-microbe symbioses, a common need is to assess the number of symbiotic microbial cells relative to the number of host cells or mass of host tissue. For example, in studies of cnidarian-dinoflagellate symbiosis, this need arises both during studies of symbiosis establishment, where the uptake rates of particular *Symbiodinium* strains may be of interest, and during studies of symbiosis breakdown (as in coral bleaching), where reliable detection of small changes in algal numbers is likely to be essential for analysis of the critical early stages in the process. It is obviously essential that the methods used be precise (*i*.*e*., highly reproducible from count to count, as indicated by low standard errors). It is also desirable, although for many purposes less critical, that the methods used be accurate (*i*.*e*., yield numbers close to the true values). And it is also highly desirable that the methods not be excessively tedious, so that large numbers of samples can be processed reliably in a reasonable amount of time.

Many studies of cnidarian-dinoflagellate symbiosis have used hemocytometer counts (or similar manual visual counts) on tissue homogenates [1–5]. Hemocytometers are inexpensive, require very little maintenance, and allow visual confirmation that each object being counted is a cell of the appropriate type. However, this method also has serious limitations. Hemocytometer counts have low precision due to random error and the relatively small numbers of cells counted, even when many replicate counts are made (reviewed by [2,6]). In addition, counts are frequently inaccurate due to systematic errors [2,6–9]. Indeed, even when used properly, hemocytometers are only accurate within ±20% of the true count [2,7,10]. Finally, the counting itself is mind-numbingly tedious, particularly when maximal precision is sought by counting large numbers of replicate samples.

A few studies have attempted to improve on the precision, accuracy, and/or time-effectiveness of manual visual counts by using cell-imaging software [3,11,12]. However, this approach is subject to the same potential sources of systematic error as hemocytometer counts, as well as uncertainty about the reliability of the counting software to count all of the cells of interest and only those cells.

Other studies of cnidarian-dinoflagellate symbiosis have used measurements of dinoflagellate chlorophyll and/or other pigments (by color intensity, fluorescence, or absorbance after extraction) as a proxy for *Symbiodinium* numbers [13–23]. For example, pulse-amplitude-modulated (PAM) and fast-repetition-rate (FRR) fluorometers have been widely used in field studies to measure chlorophyll-fluorescence intensities and to follow the progress of coral bleaching [15,21]. However, this approach to assessing algal numbers presumes a constant pigment content per algal cell, whereas in reality the pigment content often varies seasonally and may also respond disproportionately to changing environmental conditions [15,18,21]. Indeed, the correlation between chlorophyll content and *Symbiodinium* cell numbers can be non-existent or even negative, in that under bleaching conditions, the chlorophyll content can remain constant or even increase even though the numbers of algal cells are decreasing [13,18,20,23].

In attempts to circumvent the substantial systematic and random errors associated with the methods described above, some investigators have used sophisticated electronic counting methods. The Coulter Counter was developed for counting blood cells [24] but has also been widely used for counting microbial cells [6,25–27] including various types of algal cells [28–37]. It measures the impedance pulses produced as particles suspended in an electrolyte solution pass through a small orifice in a glass tube through which current (carried by the electrolyte) is flowing between internal and external electrodes [24,26,38]. As the impedance pulses are proportional to particle volume, the instrument provides information about particle volumes as well as numbers; as large numbers of cells are typically counted, the intrinsic precision is high.

In addition, several types of flow cytometer have also been used to quantify microbial cells including algae [5,32,39–42]. As a stream of particles passes through the instrument, individual particles pass a point of laser illumination, which allows a measurement for each particle of both light scattering (related to cell size and refractive index) and fluorescence. Thus, the endogenous chlorophyll fluorescence of algal cells can be used to help discriminate them from non-algal cells and debris in the cell suspensions. Again, large numbers of cells can be counted, providing a high intrinsic precision to the counts, and automated instruments can potentially result in a savings in user time [43]. However, many flow cytometers are expensive to purchase and/or use, and many do not offer effective automated handling of samples, so that extensive operator time is required to process a large number of samples.

Finally, a few recent studies have used quantitative PCR (qPCR) on algal and host genes to assess the relative amounts of each partner and how those amounts change over time [3,4,44–46]. Although this approach has clear advantages for certain purposes, it also has significant limitations, as considered further in the Discussion.

In the course of our efforts to develop the small sea anemone *Aiptasia* as a model system for studies of cnidarian-dinoflagellate symbiosis, we have systematically evaluated various methods for quantifying *Symbiodinium* cells either in culture or as isolated from anemone or coral host tissue. We have examined the efficacy of several methods of sample preparation and storage before counting, as well as the precision, accuracy, and time-effectiveness of several cell-counting methods. We found that for most–although not all–purposes, automated cell counting with the non-sorting Guava EasyCyte flow cytometer (Millipore) and normalization of the cell counts to total protein measurements offered the optimal combination of high precision, seemingly high accuracy, an ability to count cells over a wide range of concentrations, and minimal operator time.

## Materials and Methods

### Organisms

Dinoflagellates of the clonal, axenic *Symbiodinium* strains SSA02 and SSB01 [47] were cultured in IMK medium at 24–26°C with lighting on a 12 h:12 h light:dark cycle using white fluorescent bulbs (Philips ALTO II 25W) at irradiances of 10–25 μmol photons m^-2^ s^-1^ of photosynthetically active radiation as measured using a GMSW-SS quantum meter (Apogee) [47]. Anemones of the clonal *Aiptasia* lines CC7 and H2 [47,48] were cultured at 24–27°C in artificial sea water (ASW) prepared using Coral Pro Salt (Red Sea, Houston) at 33.5 ppt in deionized water (dH_2_O); lighting was as described for the algal cultures. Anemones were fed every 2–3 d with freshly hatched *Artemia* nauplii followed by water changes. Aposymbiotic anemones were prepared and maintained as described previously [47]. To anesthetize anemones, we added 3–4 drops of saturated menthol solution in ASW to ~5 ml of ASW containing one or more anemones [49]; after ~10 min, the anemones were relaxed, with tentacles fully extended, and no longer responded to tactile stimulation.

The coral samples used were nubbins of *Pocillopora damicornis* that had been cultured in an indoor aquarium system at 26°C under ~700 μmol photons m^−2^ s^-1^ irradiance on a 12 h:12 h light:dark cycle. To examine possible loss of algae under heat stress, some nubbins were moved to 33°C for 3 d under the same light conditions, while control nubbins remained at 26°C. Fragments of each nubbin were then frozen at -20°C until processing.

### Preparation of samples

After various preliminary experiments (see below), we settled on the following standard protocol for use with anemones. Individual animals are transferred into dH_2_O containing 0.01% SDS detergent (Sigma-Aldrich) and frozen in a small volume of that solution. For processing, animals are thawed and then homogenized using a two-step protocol. First, a PowerGen125 rotor stator (Fisher Scientific) is used at its highest setting (30,000 rpm) for 8–10 s. The sample is then needle sheared by passage five times through a 25-gauge needle affixed to a 1-ml syringe. Using this protocol, no visible anemone tissue remains, but the algal cells remain intact and are well dispersed. Any necessary dilutions are also made into dH_2_O with 0.01% SDS.

In some experiments (including those used in developing the standard protocol), we varied the procedure in one or more of the following ways. (i) ASW was used instead of dH_2_O. (ii) Fresh anemones were homogenized without prior freezing and thawing. (iii) The SDS was omitted or used at a higher concentration. (The actual concentration proved to matter little or not at all–see Results.) (iv) Homogenates of fresh anemones prepared using the rotor stator (see above) were fixed with formaldehyde before further processing. In this case, after homogenization in ASW, we added formaldehyde in ASW to a final concentration of either 0.7%, with subsequent incubation at ~24°C for 4 h (Coulter-Counter experiment), or 3.7%, with subsequent incubation at 4°C for 30 min and ~24°C for 1 h (flow-cytometer experiment); controls received the same volumes of ASW without formaldehyde and were processed further without the subsequent incubations. After addition of 1% SDS in ASW to a final concentration of either 0.167% (Coulter-Counter experiment) or 0.09% (flow-cytometer experiment), the samples were needle-sheared as described above. (v) Anemones were homogenized manually until no visible tissue remained using a Kontes Duall 20 Teflon + glass tissue homogenizer instead of the rotor stator; no subsequent needle shearing was performed.

To prepare samples of algal culture for counting, samples were diluted 10-fold with 0.01% SDS in dH_2_O (Coulter-Counter experiment) or 3-fold with 0.1% SDS in ASW (flow-cytometer experiment) and needle sheared as described above.

To analyze coral samples, tissue was removed from the skeleton using a single-action siphon-feed airbrush (Paasche) filled with ASW. The resulting tissue slurry was homogenized with the rotor stator (as above), needle sheared (as above but using an 18½-gauge needle), and allowed to stand for ~2 min to allow skeletal fragments to settle. The supernatant was transferred to a new tube and further needle-sheared using a 25-gauge needle until no tissue was visible. An aliquot was frozen at -20°C for later protein analysis, and the remaining sample was centrifuged at 7,000 x *g* for 5 min. The supernatant was removed, and the algal pellet was resuspended in 0.01% SDS in dH_2_O using a 25 G needle and 1-ml syringe.

### Measurements of total protein in homogenates

When appropriate, algal counts were normalized to total protein of the corresponding homogenates as determined using either the Thermo Scientific Pierce BCA assay (Fisher) or the DC protein assay (Bio-Rad). Both assays are compatible with solutions containing SDS. Standard curves for both assays were generated using serial dilutions of bovine serum albumin (BSA) in 0.01% SDS in dH_2_O.

### Determinations of algal numbers using a hemocytometer

10-μl aliquots of appropriately diluted anemone homogenate were added to each chamber of a 0.1-mm-deep Improved Neubauer Hemocytometer (Fisher Scientific). Cells were either counted immediately or photographed for later evaluation by using the bright-field mode of a Leica MZ16 FA stereomicroscope and a Leica DFC 500 digital camera. The four corner squares (each 1 mm^2^) of each 3 x 3-mm chamber were counted, for a total of eight counts. The procedure was repeated, and the 16 counts were averaged.

### Determinations of algal numbers using the Dinofinder image-analysis program

We developed the Dinofinder program to distinguish and count algal cells by user-defined pixel color intensity in microscopic images. In practice, anemone homogenate is prepared (and diluted if necessary) in a low-salt solution in order to avoid crystal formation during the drying step. Approximately 20 1-μl aliquots, each containing 10–100 algal cells, are spotted on cleaned and uncoated glass microscope slides and allowed to dry for ≥1 h while protected from light and dust contamination. The endogenous chloroplast fluorescence is then visualized and photographed using a fluorescence stereomicroscope (Leica MZ16 FA or equivalent) under blue light (GFP filter set), with the magnification adjusted such that each spot fills the entire frame. Images are processed with the Dinofinder plug-in in ImageJ (http://rsbweb.nih.gov/ij/) using the default settings (see S1 Text).

### Determinations of algal numbers using a Coulter Counter

A Coulter Counter Z2 (Beckman Coulter) was used with a 100-μm aperture and Z2 AccuComp v3.01a software. Except where indicated, samples were diluted ≥20-fold into Isoton II electrolyte solution in Acuvette cuvettes (Beckman Coulter) before counting, and the instrument was set to detect particles of 6–14 μm with a 500-μl metering volume. (The impedance pulses detected by the instrument are proportional to particle volumes. However, in the Coulter Counter documentation and literature, particle size is customarily indicated as a linear dimension, namely the diameter of a presumed perfect sphere of that volume. We also follow that practice here.) To create a particle-size-distribution profile, data from the Z2 were imported into Prism6.0a (GraphPad Software).

### Determinations of algal numbers using the Guava flow cytometer

A Guava easyCyte HT 2-laser flow cytometer (Millipore) was used with excitation by the blue (488-nm) laser and the gain controls at their default settings (yellow and green fluorescence) or set to 9.93 (forward scatter), 4.0 (side scatter), and 3.51 (red fluorescence). Samples of 200–250 μl were analyzed in 96-well round-bottom plates (Corning Life Sciences) with automatic mixing of each well for 7 s at high speed before sampling. Algal cells were discriminated from host cells and debris by their combination of side scatter (reflecting both their size and refractive index) and red fluorescence using the InCyte v2.2 software (Millipore).

### Data analysis

The data generated in each experiment were imported into Prism 6.0a (GraphPad Software). Replicates were averaged and the standard errors of the means were calculated.

## Results

### Determination of total protein as a measure of tissue mass

**Fig 1:**
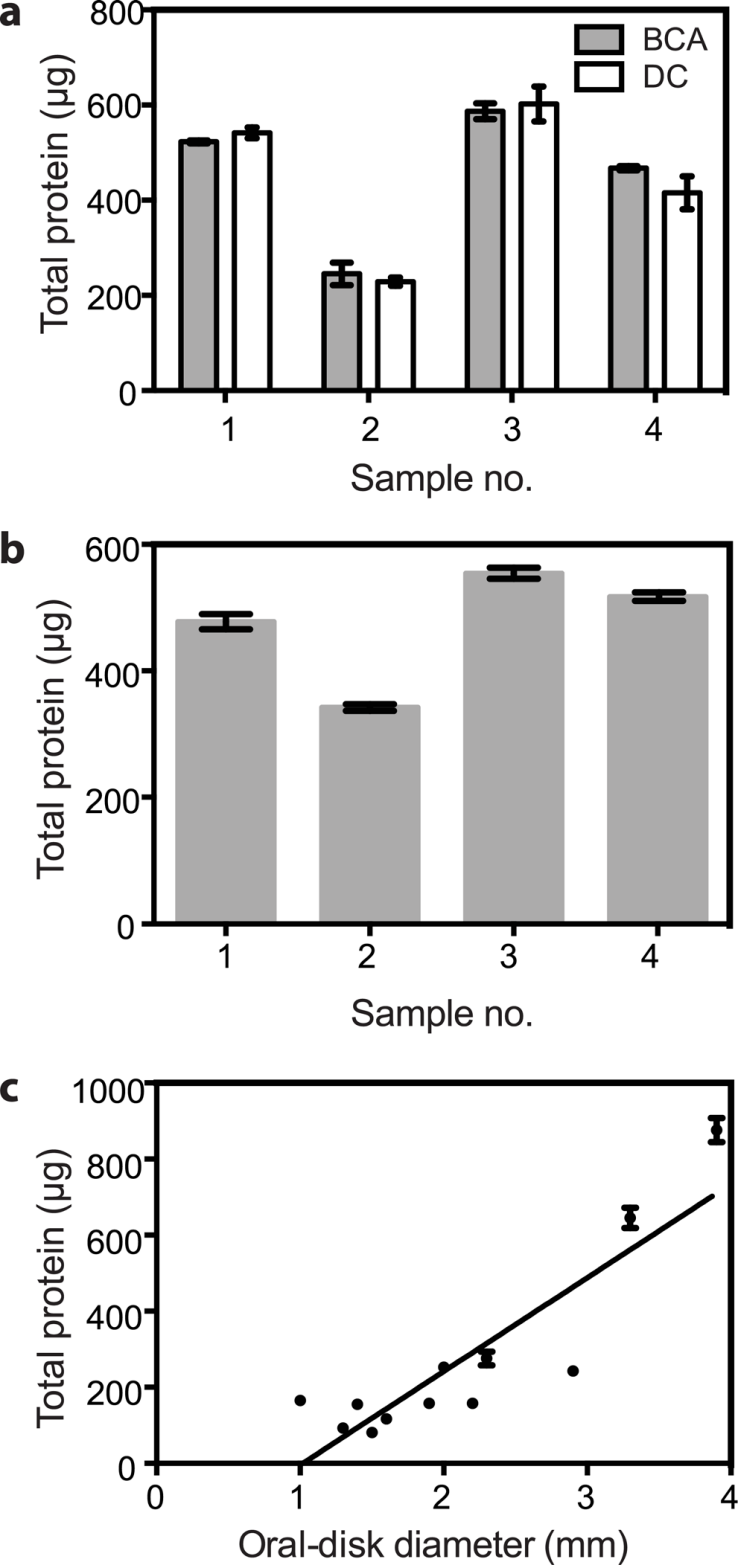
Reliability of total-protein determinations as a measure of total anemone tissue mass. Symbiotic anemones of strain H2 were homogenized and total protein was measured using our standard protocols (see Materials and Methods). (a) Comparison of the BCA and DC SDS-compatible protein assays. The total protein in each of four separate homogenates was determined with each assay. Means ± SEMs (n = 3) are shown. (b) Precision of the BCA assay. For each of four separate homogenates, total protein was measured repeatedly with the BCA assay. Means ± SEMs (n = 6) are shown. (c) Correlation between oral-disk diameter and total protein. Twelve anemones of various sizes were anesthetized (see Materials and Methods) and then photographed using bright-field illumination on the stereomicroscope to measure their oral-disk diameters. Each anemone was then homogenized and its total protein determined in triplicate using the BCA assay. Means ± SEMs are shown. R^2^ = 0.7765 for the correlation shown.

Counts of symbiotic algae typically need to be normalized to some measure of the amount of holobiont tissue from which the algae were derived. The morphological complexity and plasticity of many hosts renders measurements of their linear dimensions or volumes difficult and/or unreliable, but total protein should be a reliable measure of total tissue mass. Thus, we investigated the BCA and DC protein assays (see Materials and Methods), both of which are advertised as compatible with the SDS detergent that we found to be important to disperse cells for automated counting (see below). Both methods appeared to be both precise, as judged by the small standard errors observed, and accurate, as judged by their close agreement with each other (Fig 1a and 1b). Not surprisingly, there existed a positive correlation between total protein and anemone size as measured by oral-disk diameter (Fig 1c).

### Limitations of manual and automated microscopic counts of algal numbers

Direct microscopic counts of *Symbiodinium* in fixed larvae [50–53] or living adult anemones are possible using a fluorescence stereomicroscope, and they have the advantages (i) of not requiring sacrifice of the adult animals, so that the same animals can be scored repetitively over multiple days, and (ii) of allowing counts under circumstances (such as during the early stages of infection) when algal numbers are too low to allow reliable counting by other methods. However, we have found such counts to be challenging at algal numbers greater than ~20 per larva or ~30 per adult animal (particularly with larger anemones, where the presence of algae in multiple focal planes makes it difficult to keep track of individual cells, even in anemones that have been anaesthetized using menthol or MgCl_2_), and it appears impossible to apply this method reliably to any cnidarian producing a calcareous skeleton. In addition, there is no good way to normalize such counts to a measure of adult anemone size without sacrificing the animal and thus also the advantages of repetitive counting (although such sacrifice can, of course, be performed at the end of an experiment).

**Fig 2:**
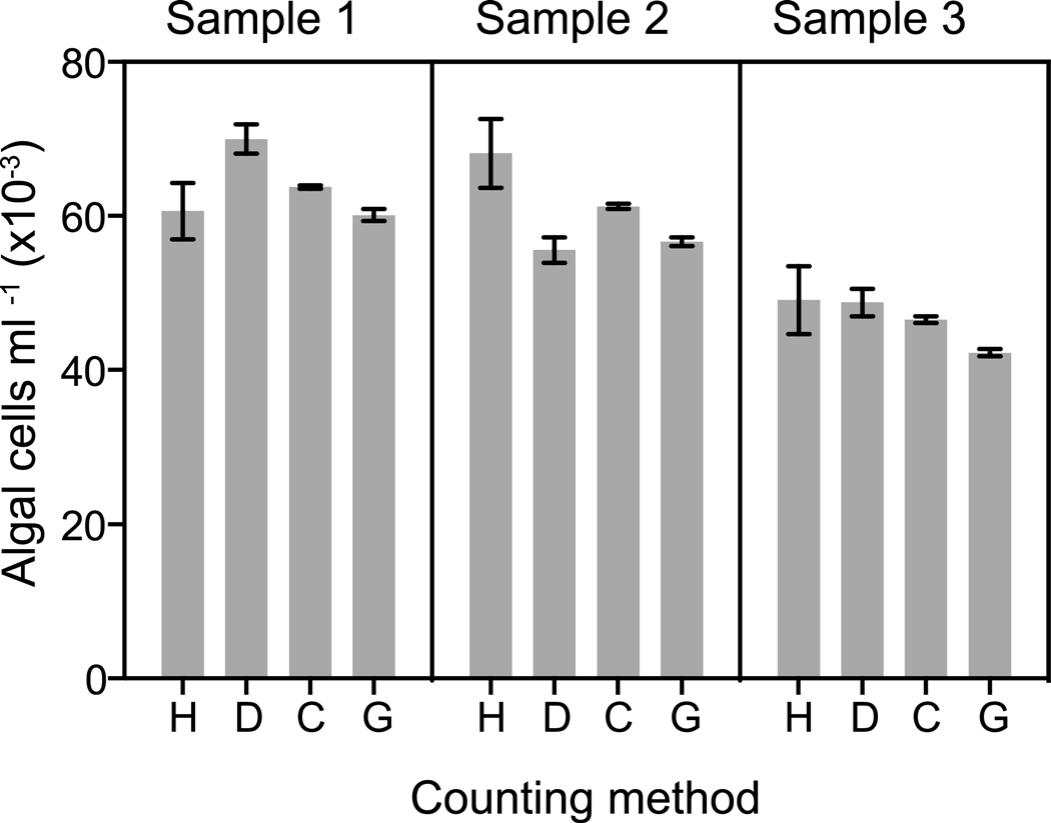
Differences in precision of different methods for counting algal cells. Symbiotic anemones of strain CC7 were washed in ASW and suspended in a small volume of solution containing one part ASW, 7 parts dH2O, and ~0.08% SDS. The animals were then homogenized in a manual tissue homogenizer (see Materials and Methods), and Samples 1, 2, and 3 were prepared by further dilution of the homogenate with the same solution. The concentration of algal cells in each sample was then determined using a hemocytometer (H), the software program Dinofinder (D), the Coulter Counter (C; particles from 6.5–12 μm were scored), and the Guava flow cytometer (G). In this case, the Coulter Counter samples were further diluted and counted in filtered ASW rather than Isoton as described in Materials and Methods. The means ± SEMs of replicate counts by each method are shown (H, n = 16; D, n = 16; C, n = 4; G, n = 8). (Note that for the hemocytometer, each one of the n = 16 itself represented an averaged count of 16 individual 1 x 1 mm squares–see Materials and Methods and Table 1.)

Manual counts of algal numbers in homogenates using hemocytometers have been very widely used but suffer from inherently low precision and accuracy (see Introduction and Fig 2, lanes H) and require large amounts of investigator time (Table 1, line 1). We attempted to circumvent these limitations by developing the "Dinofinder" automated counting algorithm, which detects the algae based on their size and autofluorescence intensity (see Materials and Methods and S1 Fig). Dinofinder indeed improved the precision of counts significantly (Fig 2, lanes D), but it was unclear whether accuracy was also improved. In addition, use of Dinofinder with sufficient care to realize the potential improvement in precision proved to require at least as much time as manual counting (Table 1, line 2).

**Table 1:** Preparation and processing times for algal-counting methods.

			Operator time for counting (min)^b^		
Line	Method	Instrument preparation time (min)^a^	1 sample	16 samples	96 samples
1	Hemocytometer	1	6^c^	96	576^d^
2	Dinofinder	1	10	160	960^d^
3	Coulter Counter	16^e^	1.5	24	144
4	Guava flow cytometer	12	0.25	0.5^f^	3^f^

^a^The time required for the user to prepare for counting, independent of the number of samples to be counted. Based on multiple trials in our laboratory.

^b^The time actually expended by the user, independent of machine-running time for the Guava. Based on multiple trials in our laboratory.

^c^Counting 16 1x1 mm squares. The four corner squares were counted in each chamber. Thus, two loadings of each sample were performed, which increases the time required for counting but decreases the chance of being misled by a poor loading of the chamber (one of the well known sources of systematic error with the hemocytometer).

^d^On the rather unlikely assumption of an indefatigable user.

^e^Preparation time can be considerably longer if the instrument clogs.

^f^Using a multichannel pipet to load the wells of a 96-well plate.

### Precise determinations of algal cell numbers using the Coulter Counter

**Fig 3:**
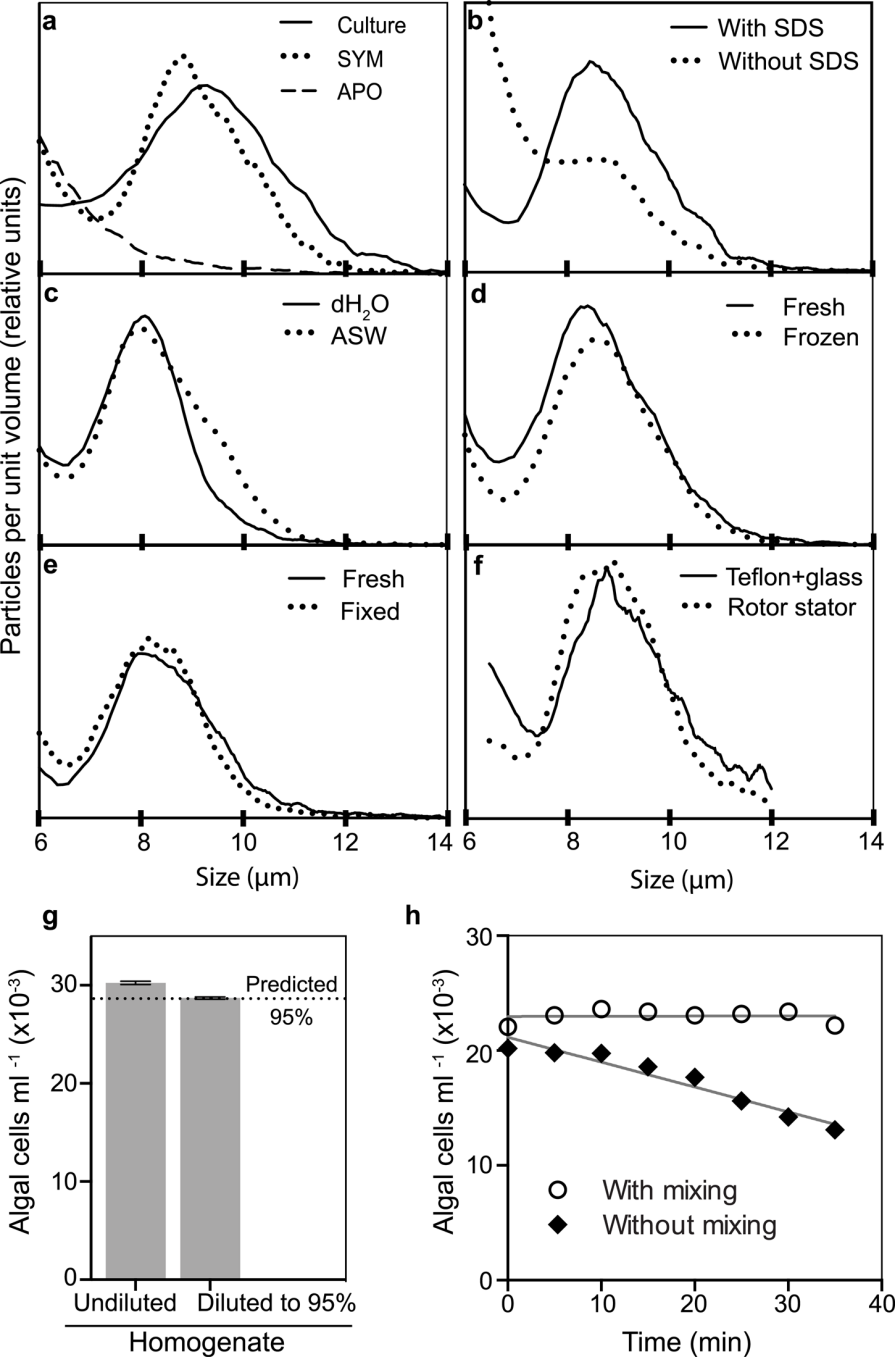
Quantification of algal cells using the Coulter Counter. All samples except those in f and h were diluted into Isoton before counting (see Materials and Methods). (a-f) Particle-size distributions. (a) A sample of cultured *Symbiodinium* strain SSA02 (Culture) was prepared as described in Materials and Methods, and homogenates of symbiotic (SYM) and aposymbiotic (APO) anemones were prepared using our standard protocol but with ASW rather than dH_2_O. (b) Fresh (*i*.*e*., not previously frozen) anemones were homogenized in ASW using the rotor stator, and samples were mixed one-to-one with either ASW or 10% SDS in ASW and needle-sheared. (c) Fresh anemones were homogenized in dH_2_O or ASW using the rotor stator, mixed with one-fifth volume of 1% SDS in dH_2_O or ASW, respectively, and needle-sheared. (d) Anemones were homogenized (rotor stator plus needle-shearing) in 0.1% SDS in ASW with or without prior freezing and thawing. (e) Anemone homogenates were prepared with or without formaldehyde fixation after homogenization as described in Materials and Methods. (f) Fresh anemones were homogenized in ASW containing 1% SDS using either the rotor stator or a manual tissue homogenizer (see Materials and Methods); the homogenates were diluted 20-fold with filtered ASW before counting. (g) A homogenate was prepared using our standard protocol, and the algal concentrations were measured in both an undiluted sample and a sample diluted to 95% of the original concentration. Gray bars, means of four replicate measurements; black lines, SEMs (too small to be resolved on this scale); dotted line, predicted 95% value. (h) A homogenate of fresh anemones was prepared in ASW using the manual tissue homogenizer (see f). One-tenth volume of 1% SDS in dH_2_O was added, the sample was diluted ~60-fold into ASW, and 10-ml aliquots were placed into two Coulter-Counter cuvettes. Each sample was mixed thoroughly and counted (t = 0). At 5-min intervals thereafter, each sample was counted again, in one case with no further mixing and in the other case with a thorough mixing prior to each count. Mixing was performed carefully to avoid introducing air bubbles. R^2^ values for the regressions shown are 0.001 (with mixing) and 0.94 (without mixing). In g and h, particles of 6.5–12 μm (*cf*. the plots in a-f) were included in the counts.

The Coulter Counter has been used for many years for precise, accurate, and reasonably rapid counts of both blood cells and various types of microbial cells (see Introduction). To assess its use with *Symbiodinium*, we first determined the particle-size distribution of *Symbiodinium* cells using an axenic cultured strain. As expected [54,55], the profile had a peak at ~10 μm (Fig 3a). A similar population of particles was detected in a homogenate of symbiotic anemones but not in one of aposymbiotic anemones (Fig 3a), and fluorescence microscopy confirmed that the aposymbiotic homogenate was indeed free of algal cells with chlorophyll fluorescence (S2a Fig).

Three potential problems with use of the Coulter Counter on samples of algae derived from host tissue are (i) that the algal cells might clump (with or without associated host-derived debris) and thus not be counted properly, (ii) that intact host cells or clumps of host-derived debris of the proper size would be mistakenly included in the count of algal cells [32,56], and (iii) that large clumps of debris might clog the orifice, resulting in operator time lost to unclogging and repeating counts. It seemed likely that all of these problems could be avoided by including detergent in the homogenization mixture, and indeed we found early in our studies that detergent was essential to reduce the background and reveal a well defined peak of algal cells (Fig 3b). Microscopic examination confirmed that the detergent treatment produced a well dispersed suspension of intact algal cells with little other particulate matter visible (S2a Fig). Although early experiments used a variety of SDS concentrations (Figs 2 and 3), the actual concentration appeared not to matter over quite a wide range, and we settled on 0.01% as the concentration for our standard protocol.

Because the Coulter Counter detects particles by their effects on electrical conductance, we compared the results obtained after processing anemones initially in dH_2_O or ASW. We observed little or no difference in the particle-size profiles (Fig 3c), presumably because the dilution into the manufacturer-supplied ISOTON II electrolyte solution was sufficient to mask any differences in initial conductivity.

As it is not always feasible to process and count samples immediately, we asked if samples could be frozen or fixed to preserve them for later analysis. When we froze intact anemones and then thawed them before homogenization and counting, the particle-size profile was essentially the same as that obtained after homogenizing fresh anemones (Fig 3d). In contrast, when we froze homogenates and thawed them just before counting, we observed an increase in cell debris and background counts that was sufficient to make the samples unusable (data not shown). Fixation of homogenates with formaldehyde also proved to be an effective way to preserve samples for later analysis (Fig 3e). In contrast, when we fixed intact anemones with formaldehyde, subsequent homogenization (at least by our standard procedures) produced inhomogeneous suspensions that yielded inaccurate counts and frequently clogged the Coulter Counter aperture (data not shown).

Homogenization of anemones using the PowerGen rotor stator (as in our standard procedure) was easy and rapid (8–10 s per sample of one or several anemones), but it seemed possible that it would either over- or under-homogenize the samples. However, samples processed in this way yielded particle-size profiles essentially identical to those produced more laboriously (~1 min per sample) using a conventional manual homogenizer (Fig 3f). The rotor stator was also significantly easier to clean between samples than the manual homogenizer.

As expected, the Coulter Counter produced counts of high precision that could easily discriminate between samples differing in algal concentration by as little as a few per cent (Fig 2, lanes C; Fig 3g).

Another concern about use of the Coulter Counter was that precision and/or accuracy might be lost because of settlement of the algal cells prior to counting. Indeed, when we allowed a sample to sit without mixing for up to 40 min before counting, the counts declined progressively (Fig 3h). However, when a parallel sample was mixed before each count, this problem was totally avoided (Fig 3h).

Finally, we found that not only did the Coulter Counter allow significantly more rapid counting than did the hemocytometer (Table 1, line 3), but also that the alternating routine of mixing, loading, and short breaks while the machine performs the counts made the process more sustainable over long periods. In a long series of counts, some time is unavoidably lost to the need to deal with the occasional clogging of the orifice, but we did not find that to be a significant problem in a reasonably clean laboratory setting.

### Precise and rapid determinations of algal cell numbers using the Guava flow cytometer

**Fig 4:**
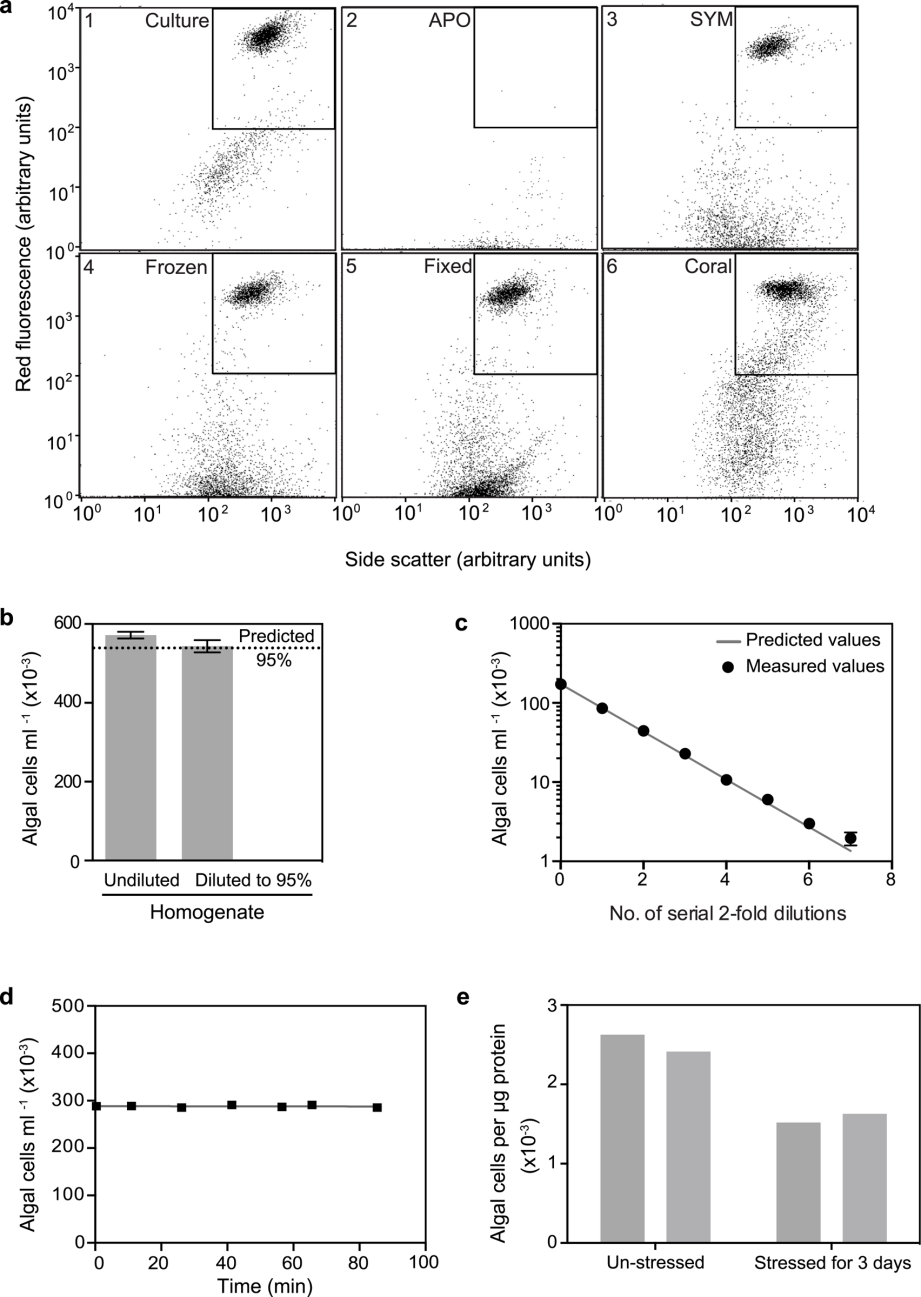
Quantification of algal cells using the Guava flow cytometer. The instrument was operated as described in Materials and Methods; all anemones were of strain CC7. (a) Effects of sample type and preparation method on the flow-cytometer plots of the red (chlorophyll) fluorescence vs. the side-scatter of the particles. The square within each plot indicates the region containing *Symbiodinium* cells with approximately normal chlorophyll concentration and light scatter. The samples of cultured algae (strain SSA02), fixed anemone homogenate, and coral homogenate (panels 1, 5, and 6) were prepared as described in Materials and Methods. The homogenates of aposymbiotic (APO) and symbiotic (SYM) anemones (panels 2 and 3) were prepared using our standard protocol except (i) the anemones had not been frozen and (ii) the symbiotic anemone was initially homogenized by rotor stator in ASW, after which one-fifth volume of 0.1% SDS in ASW was added before needle shearing and further dilution in 0.1% SDS in dH_2_O. The frozen anemones (panel 4) were frozen in 0.1% SDS in ASW, then thawed, homogenized by rotor stator and needle shearing, and diluted further in the same solution. (b and c) Precision of the method and its ability to detect small differences in algal-cell concentrations. Anemone homogenates were prepared and diluted using our standard protocol. (b) Undiluted and diluted homogenates were analyzed as in Fig 3g except that n = 8 in this case. (c) Serial two-fold dilutions of the homogenate were prepared, and the predicted (solid line) and measured (as mean values ± SEMs; n = 3) counts were compared. Most error bars are hidden by the points themselves with the graphing program used here. (d) Efficiency of automatic mixing by the instrument. Anemone homogenate was prepared by our standard protocol except that 0.1% SDS in ASW was used for homogenization and dilution. Samples were added to all wells of a 96-well plate, which was then analyzed in the usual way, so that individual samples sat for up to 84 min without agitation except for the automatic mixing that preceded the sampling from each well. R2 for the regression shown = 0.04. (e) Quantification of algal cells in two coral nubbins that had been held at 26°C and in two nubbins that had been stressed at 33°C for 3 d (see Materials and Methods). Homogenates were prepared as described in Materials and Methods, and algal cell numbers (see a, panel 6) were normalized to protein concentrations as determined by the BCA assay (see Materials and Methods).

In seeking a method that offered both high precision and rapid, automated processing of samples, we tested the Guava flow cytometer, which detects and counts particles passing through a microcapillary tube on the basis of their fluorescence and light scattering (www.millipore.com/easycyte); this method seemed likely to work well with *Symbiodinium* cells given their intrinsic chlorophyll fluorescence. Indeed, analyses of both axenic *Symbiodinium* cultures and homogenates of symbiotic anemones revealed a cluster of particles with high red fluorescence and a well defined range of light-scattering values (Fig 4a, panels 1 and 3). Such a population was totally absent in a homogenate of aposymbiotic anemones (Fig 4a, panel 2) and can be bracketed for automated counting by the instrument (inset boxes in Fig 4a). Although the precision of such Guava counts was slightly less than that obtained with the Coulter Counter (Fig 2, lanes C and G), it was still far superior to that obtained with the hemocytometer (Fig 2, lanes H and G), and it was sufficient to reliably detect small changes in algal numbers over a wide range of algal concentrations (Fig 4b and 4c). The Guava also requires much smaller sample sizes than does the Coulter Counter (Table 2), and it is much faster to use than either the Coulter Counter or hemocytometer, particularly when large numbers of samples are to be counted (Table 1, line 4). In addition, the automatic mixing that occurs before sampling from each well of the 96-well plate in the Guava means that settlement of the algal cells is not a problem (Fig 4d). This last experiment also shows that any gradual dissolution of the algal cells by the SDS used and/or by autolysis is not a problem on the timescale in which the counts are performed.

**Table 2:** Summary of the properties of the counting methods.

Method	Optimal algal concentration range^a^ (cells ml^-1^ x10^-3^)	Required sample volume^a^ (ml)	High-throughput capability	Operator time	Common sources of problems	Potential for human error
Hemo-cytometer	200–600	0.04	No	High	Clumps, sample volume, loading error	High
Dinofinder	10–100	0.016	No	Very High	Debris, high salt concentration, clumping	High
Coulter Counter	100–1,000^a^	6	No	Moder-ately high	Incomplete homogeniza-tion, clumping	Low
Guava flow cytometer	10–500	0.1	Yes	Low	Clumps	Low

^a^At the time of counting. Note that in our standard procedure, the sample is homogenized in 500 μl and then diluted (if needed) to the optimal concentration for counting. This dilution step is not required except for samples to be counted with the Coulter Counter, for which a dilution of ~20-fold into Isoton or some comparable solution is needed. Thus, for counting with the Coulter Counter, the algal concentrations in the original homogenate must be correspondingly higher.

As with the Coulter Counter, we found that intact anemones could be frozen and stored, and later thawed and homogenized, without appreciably affecting the fluorescence/scatter profile subsequently observed (Fig 4a, panel 4), and that formaldehyde fixation of homogenates also did not appear to affect the population of cells in the counting window (Fig 4a, panel 5). Fixation did appear to increase the number of nonfluorescent particles with light-scattering similar to that of *Symbiodinium* cells (Fig 4a, panel 5), possibly because of the stabilization of clumps of material with sufficient size and density to give such scattering, but there was not enough of this material in the counting window to represent a significant problem for obtaining reliable counts. However, when we split homogenates in half, fixed one of each pair of samples, and compared the counts, we found that the fixed samples consistently gave counts that were 10–30% lower than the samples that had not been fixed (data not shown). The reason for this difference is not known, and it is possible that the use of a different fixative such as glutaraldehyde would avoid this problem.

The number of particles that had little or no red fluorescence but scattered light like *Symbiodinium* cells was correlated with the presence of *Symbiodinium* cells themselves (Fig 4a, panels 1–3; S2b Fig), raising a concern that this material represented *Symbiodinium* cells with reduced fluorescence, so that not including them in the counting window would give misleading results. However, careful comparison of DIC and fluorescence microscopy images of cell culture and of homogenates of symbiotic and aposymbiotic animals revealed no nonfluorescent bodies that resembled algal cells in size and shape (S2a Fig). Thus, we infer that although this debris appears to be algal-related, it is not actually algal cells, and thus does not needed to be included in the counts.

As our goal in these studies was to assess methods for their utility not only in the *Aiptasia* model system but also with corals, we were concerned that residual skeletal fragments in coral homogenates might clog or break the microcapillary tube in the Guava. However, a simple procedure to eliminate such fragments (see Materials and Methods) allowed coral samples to be processed without incident (Fig 4a, panel 6; [22]). Moreover, the Guava counts were of sufficient precision to detect small changes in the numbers of algal cells per unit of holobiont protein during heat stress leading to bleaching (Fig 4e).

## Discussion

The breakdown of cnidarian-dinoflagellate symbiosis under stress is a topic of intense interest as coral reefs face the challenges of climate change and other anthropogenic stresses. Maximally effective study of this process–as well as of other aspects of the biology of the symbiosis–depends on the ability to make large numbers of highly precise measurements of the numbers of dinoflagellate cells relative to the amounts of holobiont tissue. Although the hemocytometer has been widely used for this purpose, its well known limitations (see Introduction) prompted us to seek a better method by using the *Aiptasia* model system to systematically compare the precision and speed of counting of several alternative methods relative to those of the hemocytometer. As discussed in more detail below, we found that for most purposes, the Guava flow cytometer offers the most attractive constellation of characteristics (Table 2), and we showed that it can also be used with samples of hard corals.

### Pros and cons of the counting methods evaluated here

As expected, we were able to achieve only modest precision with the hemocytometer even when doing multiple replicate counts. As the SEMs were ~7% of the means, small changes in algal numbers (as during the initial stages of bleaching) would be impossible to detect reliably, a major limitation for attempts to elucidate bleaching mechanisms. In addition, such manual counting requires large amounts of operator time, and we think it almost inevitable that the frequency of human errors (further undercutting the reliability of the counts) will increase as operator exhaustion sets in. Nonetheless, the hemocytometer clearly will continue to have appropriate uses, such as when only one or a few samples are to be counted (Table 1) and high precision is not needed, or when more sophisticated instruments are not available.

We attempted to improve upon the hemocytometer by developing the Dinofinder software for computerized recognition and counting of cells with chlorophyll fluorescence after spotting samples onto microscope slides. When used carefully, Dinofinder yielded a significant increase in precision over manual counts. However, realizing this high precision required a very high level of care in sample preparation, dilution, and loading, with a corresponding high potential for human error, and we were unable to process samples even as rapidly as with the hemocytometer. It is possible that additional effort could have mitigated these problems and improved the utility of this approach, but there was little incentive to do so once the potential of the Coulter Counter and Guava flow cytometer became clear. Thus, at present, we think that the greatest utility of Dinofinder (or a similar counting program) might be during field work in a situation in which there is no way to count samples immediately after collection: slides made in the field can be counted at a later time in the laboratory, as the algal autofluorescence remains detectable for at least several weeks after slide preparation.

In contrast, the Coulter Counter provided both a dramatic improvement in precision, due in part simply to the large numbers of cells counted in each sample, and an ~4-fold savings in operator time (and reduced tedium) when counting multiple samples. The instrument can potentially over-count (if intact host cells or clumps of debris are mistakenly counted as *Symbiodinium* cells) or under-count (if samples are not well mixed, if small clumps of cells are counted as single cells, or if the algal-cell concentration is too high, such that there is significant coincidence of two or more cells passing through the orifice at the same time). However, we found that none of these potential problems actually arose when we homogenized samples well in the presence of a low concentration of detergent and took care to mix each individual sample well immediately before counting. Despite its virtues, the Coulter Counter also has some significant limitations including its moderately high purchase price (currently ~$18,000 for a basic instrument), the fact that it performs best in a clean and stable laboratory environment (and thus is unlikely to be deployed in the field), the substantial (and essentially continuous) operator time involved in counting a large number of samples with this non-automated instrument, and (the most significant problem in our experience) its requirement for large sample volumes at relatively high algal-cell concentrations (Table 2).

Considering all properties together (Table 2), the Guava flow cytometer appears to offer the most attractive option for counting *Symbiodinium* cells in most applications: its precision is high, although perhaps slightly less than that of the Coulter Counter; it can effectively count algal cells over a wide range of cell concentrations and requires only a very small volume of cell suspension to do so (Table 2); and (most important) its automated processing (including self-rinsing and mixing of samples) of 96-well plates results in both reduced opportunities for human error and enormous savings of operator time and thus an ability to count large numbers of samples (including multiple biological and/or technical replicates when desired). With simple precautions (a short sedimentation at 1 x *g*; passage through a 25-guage needle), it can be used safely and effectively with samples of hard corals (Fig 4a, panel 6; Fig 4e; [22]). A complication in some studies might be if the algal cells lose pigment (and thus chlorophyll fluorescence) under stress, causing them to drop out of the window normally gated for counting, but in our experience, this situation is easy to recognize from the two-dimensional Guava plots, and thus compensate for, when it occurs [22,47]. We were also concerned about the light-scattering material with little or no fluorescence whose presence seems to be correlated with that of *Symbiodinium* cells. However, although the nature of this material is still unclear, it does not appear to be nonfluorescent algal cells based on microscopic examination.

Thus, it appears to us that the Guava has just two significant limitations for studies of cnidarian-dinoflagellate symbiosis. First, like the Coulter Counter, it is a sensitive electronic instrument that is best operated in a clean and stable laboratory environment. However, the potential impact of this limitation on its use in field studies is at least partially mitigated by the ability to stabilize and transport samples for counting later (see below). Second, the rather high purchase price (currently ~$47,000 for the basic single-laser instrument) may put it beyond the range of many individual laboratories (although not of a group of laboratories or of an institute). In this regard, we note (i) that the departmentally purchased instrument that we use has also been used successfully by colleagues working with yeast and mammalian cells that are expressing fluorescent proteins and (ii) that the slightly more expensive (~$57,000), but more versatile, two-laser instrument may be a better choice for an instrument that will be used by a variety of laboratories. The high cost of the preventative-maintenance service contract recommended by Millipore may also be inhibitory to some potential users. However, we have not purchased the contract for several years and have needed only one service call (for less than the cost of the contract) during this time.

### Chlorophyll measurements and qPCR

We have made no systematic efforts to evaluate these methods for estimating algal numbers. However, the obvious limitations of any method based on the photosynthetic pigments (see Introduction) suggest that this approach should be used only when no better method is practicable (such as perhaps at a remote field site). In limited trials, we found that qPCR was highly reproducible in repeated measurements on the same sample but that the preparation and processing of multiple samples took ≥10 times as long as the corresponding steps for the Guava flow cytometer. In addition, converting qPCR values to algal cell numbers, or to algal cell numbers relative to host cell numbers, is fraught with difficulties involving factors such as the different efficiencies of different primers and the need to establish a standard curve for any particular cell type using some other precise and accurate measurement of cell numbers [57–59]. These problems may not apply if the goal is simply to follow relative algal quantities (*e*.*g*., during a time course of infection or bleaching), and indeed qPCR might be particularly valuable in tracking relative algal numbers when these are too low to measure reliably with a flow cytometer or Coulter Counter. Moreover, subject to the caveat about probable differences in primer efficiency, qPCR does offer the unique advantage of allowing the relative numbers of two or more *Symbiodinium* strains to be assessed in parallel, as might be important during infection by a mixed algal population or in assessing whether one of two algal strains is lost differentially during bleaching of a mixedly infected colony. The relative ease of preserving DNA samples usable for qPCR during transport back to the home laboratory might also make this approach useful during work at a remote field site.

### Apparent accuracy of the counting methods

The discussion above focused on the precision rather than the accuracy of the several methods, both because precision is generally more critical to experimental applications and because accuracy is more difficult to assess, as it requires that the "true value" actually be known. However, we note that a direct comparison of the absolute values obtained with the four methods evaluated here shows a reasonably good agreement (Fig 2), suggesting that all four methods provide a level of accuracy acceptable for most applications. In particular, the most precise methods (Coulter Counter and Guava flow cytometer) agree very well with each other, consistent with the a priori expectation that these instruments would also be accurate. The slightly higher counts obtained with the Coulter Counter could reflect some counting of non-*Symbiodinium* debris by this method, a failure to count some *Symbiodinium* cells of low fluorescence with the Guava, or both.

### Preservation of samples prior to counting

Neither the Coulter Counter nor the Guava flow cytometer is likely to be immediately at hand in most field studies. However, this does not seem to be a major limitation to their use, as we showed that either freezing whole animals or formaldehyde-fixing homogenates yielded samples that could be further processed and counted at a later time. Fixation did appear to have a nontrivial and somewhat inconsistent effect in reducing the counts obtained with the Guava (see Results; this was not tested in the same way with the Coulter Counter), and so should probably be avoided when alternatives are available.

### Normalization of cell counts

No matter how counts of algal cell numbers are obtained, it is almost always necessary to normalize them to some measure of holobiont tissue mass in order to be able to compare different samples. As the error of a quotient reflects the errors of both individual terms, it is obviously critical that the measure of tissue mass have a precision that is comparable to that of the counting method itself. Although many studies have used such parameters as polyp size, oral-disk diameter, wet weight, dry weight, or (for hard corals) surface area, it seems clear that none of these approaches can provide precision sufficient to reliably assess small changes in *Symbiodinium* population densities in small samples of host material. In contrast, the protein assays tested here, when applied to the same detergent-containing homogenates that are used for the algal counts, provide an acceptable level of precision. As these assays are simple and cheap to perform and can be read either individually with a spectrophotometer or (our own routine practice) with an automated plate reader in 96-well plate format) there seems little reason not to use this approach unless only very crude measurements are needed or fieldwork is being done under conditions in which storage and transport of samples back to a laboratory is impossible. It should be noted that if the number of algae in host tissue is actively changing during an experiment (e.g., during bleaching), this change will also affect the total protein measurement (or any other measure of total holobiont material), leading to some underestimation of the true change in algal numbers relative to the amount of host tissue.

## Supporting Information

S1 Fig Identification of dinoflagellate cells using the Dinofinder program. (a) Fluorescence stereomicrograph of a sample of strain CC7 anemone homogenate using the GFP filter set; algal cells are revealed by their red chlorophyll fluorescence. (b) Location of algal cells to be counted (white dots) after processing by Dinofinder (see Supplemental Materials and Methods).
(EPS)

S2 Fig Analysis of the light-scattering particles with little or no red fluorescence as detected by the Guava flow cytometer. (a) Apparent absence of nonfluorescent algal cells in algal culture and anemone homogenates. Cultured algae (strain SSB01) and homogenates of symbiotic and aposymbiotic *Aiptasia* (strain H2) were prepared for observation using the standard protocols described in Materials and Methods. Aliquots of each preparation were then examined by DIC and fluorescence microscopy (490 nm activation, 632 nm emission) using a Nikon Eclipse 600 FN microscope equipped with a Hamamatsu ORCA-2 CCD camera and an Apo 100X/1.40 NA oil-immersion objective. Images were captured with an exposure time of 300 ms and collected using MetaMorph software (Molecular Devices). 78 algal cells from culture and 105 algal cells from a symbiotic homogenate were detected by DIC and checked to confirm that all were indeed fluorescent. No algal cells were observed in the aposymbiotic homogenate. (b) Correlation between the presence and abundance of algal cells and the presence and abundance of light-scattering, non-fluorescent particles during a bleaching and recovery time course. Strain H2 anemones acclimated to 27°C were switched to 34°C (time 0) and held at that temperature for 6 d (top panels) before being returned to 27°C for further incubation (bottom panels). At the times indicated, homogenates were prepared from single anemones using our standard protocol and analyzed using the Guava flow cytometer.
(EPS)

S1 File Supplemental Materials and Methods. (PDF)
