# Supplementary material for: Rapid, Precise, and Accurate Counts of Symbiodinium Cells Using the Guava Flow Cytometer, and a Comparison to Other Methods
Source: PLoS One. 2015 Aug 20;10(8):e0135725. doi: 10.1371/journal.pone.0135725 (PMC4546242; doi:10.1371/journal.pone.0135725)
Supplement: S1 File — (PDF) [file pone.0135725.s003.pdf]

S1_File. Supplemental Materials and Methods

Methods for Dinofinder Software

Sample homogenization and spotting

1. Homogenize anemone in 500 µl to 1 ml of 0.01% SDS in dH_2O using a rotor-stator or Kontes tissue grinder (#20 with Teflon pestle) and needle shearing.
2. If quantifying total protein using the BCA assay, be sure to save some of the homogenate.
3. Dilute tissue homogenate to achieve 10-100 algae μl^{-1}.
    a. To calculate dilution factor, either:
        i. Estimate the algal population within the anemone, OR
        ii. When all samples are homogenized, spot 1 µl of each homogenate on a glass slide and visualize under the microscope (see below).
4. Thoroughly clean a glass microscope slide with ethanol, and spot the homogenate on the slide (at least 20-24 1 µl spots).
5. Air-dry the slide in a clean environment for at least 1 hour. You should see faint outlines from the dried spots.

Microscope

6. Power up the microscope and the fluorescent bulb.
7. Turn on blue excitation / GFP filter cube. Use 20-30x magnification, making sure the spot fills the entire viewing field.
8. Use the following settings on the microscope: gain: 8.1; exposure: 2 s; gamma: 1.29, saturation: 1.0.
9. Make sure all light is going to the camera. Take pictures using the default camera settings (24 bit pictures). Label pictures and save to a known location.

Picture processing

10. Load picture into *ImageJ* (http://rsbweb.nih.gov/ij/). Follow either of the following counting protocols (manual or automated).
    a. Manual:

i. Download the plug-in "Cell Counter"

ii. Use "Cell Counter" to manually click on dinoflagellates in each picture (dinoflagellates will appear as red dots). Record counts per picture.

b. Automated:

i. Use program Dinofinder by Matt Burriesci (details to follow)

11. Calculate dinoflagellates per anemone, taking into account statistics of picture counting, dilution factor, etc.

Instructions for using Dinofinder10

Tested on Windows XP, Vista, Mac OSX, Ubuntu 7 and later.

To install Dinofinder10:

1. Install *ImageJ* on your system from http://rsbweb.nih.gov/ij/
2. Copy the Dinofinder10_.java file into (directory_structure)/imagej/plugins folder
3. Open *ImageJ* – you may need to rightclick → run as admin on Windows Vista to allow compilation.
4. File → Open your picture
5. Plugins → Compile and Run. Then select Dinofinder10_ (after one successful run it should be available as an option in the Plugins menu next time *ImageJ* is opened).
6. Now a screen should pop up with some options, just click OK to use the default settings. This will change the original image, so make a copy or do not save the changes after the image is processed.
7. A few seconds later the image should change and have some green with blue pixels. A file called "output.txt" should be in your *ImageJ* directory and it will contain information on each identified cell. The image itself now has green on any pixels that could have been part of cells and clue where Dinofinder decided a cell was. Dinofinder also should have removed any green channel contamination in the original image (Figure S1).
8. The options in the Dinofinder dialogue box are:

   a. Minimum Cell Radius: The minimum number of pixels on each side of a center

pixel that are expected to be above background.

b. Maximum Cell Radius: The maximum for the parameter above. Together, these two allow the computer to determine the difference between a brighter dinoflagellate and two rather close dinoflagellates.

c. Minimum Red Intensity of Red Cells: This takes a number between 0 and 255. You can find the red intensities of outer fringes of cells as the first number in the "value" field of the *ImageJ* main window when you have a curser over an image before you run Dinofinder. The background is 20 to 30, so 35 works pretty well on images from the microscope used in this study.

d. Surrounding Cutoff: This takes a value x- if 0 is the center of the pixel below

    1 2 3

    4 0 5

    6 7 8

   Then the red values of at least x of the pixels 1 to 8 must be below that of pixel 0.

e. Cumulative Total: This is similar to above. It takes pixels 1-8 and draws a 3x3 grid with them, then does the surrounding cutoff value calculation for each of 1-8 and then sums the results. These two statistics allow the effects of differences in local brightness across the image to be minimized.

f. Filename Without Extension: Name of text output file with data for each cell. New data should be appended (running Dinofinder 10 times should just keep adding data to this file).

g. Filepath: Specify a particular filepath if you don't want the output file to be saved in the *ImageJ* folder.

h. Other Features:

   i. Allows regions of interest selection: Select a sub-set of pixels on the screen with the wand or selection tools before running Dinofinder.

   ii. Images can be run on stacks: Use *ImageJ* to automatically load a stack and

it will go through each image and compute.
